# Infant Mortality and Inflation in China: Based on the Mixed Frequency VAR Analyses

**DOI:** 10.3389/fpubh.2022.851714

**Published:** 2022-03-29

**Authors:** Wei Jiang, Xin-yi Liu

**Affiliations:** School of Economics, Qingdao University, Qingdao, China

**Keywords:** infant mortality, inflation, mixed frequency VAR, SDGs, China

## Abstract

Reducing neonatal mortality is an important goal in the Sustainable Development Goals (SDGs), and with the outbreak of the new crown epidemic and severe global inflation, it is extremely important to explore the relationship between inflation and infant mortality. This paper investigates the causal relationship between inflation and infant mortality using a mixed frequency vector autoregressive model (MF-VAR) without any filtering procedure, along with impulse response analysis and forecast misspecification variance decomposition, and compares it with a low frequency vector autoregressive model (LF-VAR). We find that there is a causal relationship between inflation and infant mortality, specifically, that is inflation increases infant mortality. Moreover, the contribution of CPI to IMR is greater in the forecast error variance decomposition in the MF-VAR model compared to the LF-VAR model, indicating that CPI has stronger explanatory power for IMR in mixed-frequency data. The results of the study have important implications for China and other developing countries in reducing infant mortality and achieving the Sustainable Development Goals (SDGs). Policymakers should focus on inflation as a macroeconomic variable that reduces the potential negative impact of inflation on infant mortality. The results of the analysis further emphasize the importance of price stability in the context of global inflation caused by the outbreak of the coronavirus pandemic outbreak.

## Introduction

The purpose of this article is to analyze the relationship between inflation and infant mortality in China. Neonatal mortality is an important indicator for judging the health status of a population and evaluating social wellbeing. Reducing neonatal mortality rate is an important goal of the Sustainable Development Goals (SDGs), which is “Ensure healthy lives for all ages and promote the wellbeing of all people.” For neonatal deaths, the target is set at 12 deaths per 1,000 live births. The United Nations International Children's Emergency Fund (UNICEF) notes that global neonatal mortality remains at alarmingly high levels. High infant mortality rates reduce the health of populations and undermine human capital, which in turn hinders economic progress and development. At the same time, infant mortality can be used as an indicator of population health and economic success and failure (1). Indeed, the degree of population health (infant mortality rate) is influenced by many factors, such as the economic cycle (GDP), unemployment, income, and other macroeconomic variables (2, 3). There are also other factors such as education (4), health care expenditures (5), and even differences in ethnicity that can affect the degree of population health (6).

Inflation, as a key indicator in macroeconomics, is closely related to family life, and to a large extent is a global phenomenon (7). Rising inflation leads to higher food prices, higher cost of living for households, and reduce purchasing power, which in turn have widespread effects on people's nutrition and health status (8, 9). Since the outbreak of the coronavirus pandemic, global commodity price increases have triggered varying degrees of inflation in many countries (10, 11). According to statistics, in September 2021, the year-on-year CPI in the United States, Germany, Canada, South Korea, and South Africa increased by 5.4, 4.1, 4.4, 2.5, and 5.0%, respectively, setting new highs for the CPI index in recent years. At the same time, many countries in the world are in a state of lockdown, leading to shortages of food and other materials in the market. Added with serious inflation, which has virtually has caused great harm to the health of the global population, especially children (12).

Since the reform and opening up in the 1980s, China has experienced rapid socioeconomic development, a decrease in infant mortality, and an increase in life expectancy (13). The Chinese government is actively involved in the Sustainable Development Goals (SDGs) project (14) and has made considerable efforts to monitor and reduce maternal, neonatal, and under-five mortality rates nationwide. However, according to the China Child Development Program, although the health level of children in China has improved significantly since 2010, China is still a developing country with relatively low per capita income, and the number of neonatal deaths remains high. Therefore, studying the relationship between infant mortality and macroeconomic variables in China and making corresponding initiatives based on this relationship is important to continue reducing infant mortality and improving population health in China. Moreover, past research primarily focuses on the relationship between inflation and infant mortality in developed countries such as Jamaica and some extremely poor African countries (15–17), and this problem is poorly understood in emerging market economies. While China has practically all of the characteristics of an emerging market country (18). Besides, according to the World Trade Organization (WTO) statistics, China remained at a high level in the world in 2019 in terms of neonatal deaths (in thousands) due to the peculiarities of its population size. Therefore, the relationship between infant mortality and inflation in China is worth exploring.

This paper makes several contributions: Firstly, the existing literature focuses on the relationship between macroeconomic variables and health in high-income countries or extremely poor African countries, with very little on emerging developing countries, whereas China displays practically all of the characteristics that distinguish developing countries (19). At the same time, China is also the most populous country, and reducing the number of infant deaths in China is important for the improvement of global public health problems and the achievement of the Sustainable Development Goals (SDGs). And there is still a gap in research on the relationship between macroeconomic variables and infant mortality in China. We, therefore, choose to study the effect of inflation on infant mortality in China, which has implications for emerging developing countries.

Secondly, on the one hand, previous studies on China mainly focusing on the relationship between common factors and infant mortality (e.g., economic cycle fluctuations (GDP), income, public spending, and parental education), and there are a few studies that examine the association between inflation and infant mortality. Inflation, as a measure of the cost of living for families, invariably affects all aspects of family life, including food, clothing, housing, and transportation. Therefore, exploring the causal relationship between inflation (year-over-year CPI) and infant mortality is a pioneering work. On the other hand, although there are some studies on the relationship between food inflation and child health in African countries, most of them consider only food price inflation and do not take into account inflation of other factors as well. Therefore, we selected the year-on-year value of total CPI growth in China as the subject of our study. Trying to fill the gap in existing studies by analyzing the impact of inflation on population health (infant mortality) from a Chinese standpoint.

Thirdly, traditional methods require all data to be at the same single frequency. The majority of previous research mainly focused on high-frequency factors, and it is common to convert them into low-frequency variables by aggregation, but this process may result in erroneous statistical estimates. Therefore, to explore the effect of heterogeneity of high-frequency variables on low-frequency series and to obtain more accurate results, we used an MF-VAR model that does not require any filtering process to explore the effect of inflation on infant mortality in China and to contribute to the existing literature.

The rest of this research is structured as follows: the “Literature Review” section summarizes the relevant studies. In the section “Theoretical Analysis and Research Hypothesis,” the theoretical mechanism of the relationship between inflation and infant mortality is described and the research hypotheses are presented. The MF-VAR and LF-VAR models are described in the section “Empirical Model,” and the data utilized in this paper is described in the section “Data.” Our findings are presented in the “RESULTS” section. The section “Disscussion” discusses the results, and finally, the section “Conclusion” draws conclusions and makes relevant policy recommendations. policy recommendations.

## Literature Review

Some factors influence infant mortality, and there has been a lot of studies investigating the relationship between macroeconomic variables and infant mortality. Specifically, macroeconomic variables such as poverty, unemployment, and GDP per capita are associated with child mortality (20). Paxson and Norbert (21), Cnattingius and Bengt (22) argue that in countries such as Peru, Denmark, Finland, Norway, and Sweden, economic crises increase infant mortality, while economic growth decreases it. Similar conclusions can be obtained in developing countries as well (23, 24), that is, economic growth is countercyclically correlated with infant mortality. Kim and Marta (25) argue that the association between child health and GDP per capita growth is mixed. In contrast, Ruhm (26), Dehejia, and Adriana (27) show that infant mortality decreases instead during economic recessions. using a panel dataset of 23 cities in Taiwan and a fixed-effects model, Lin (28) finds that infant and neonatal mortality rates vary countercyclically with urban unemployment in Taiwan. Ko et al. (29) using the method of multivariate logistic regression, found that low levels of paternal and maternal employment were associated with infant mortality among preterm infants. Besides, Cutler et al. (30) find that low income and low wealth lead to high infant mortality. According to Dallolio et al. (31) and others, even income inequality affects infant mortality. However, there are not many studies on the relationship between inflation and infant mortality, especially in China. And the inflation rate is of significant research value as a key indicator in macroeconomics that affects family life in all aspects by affecting aspects such as income and price levels. So, we consider the CPI, to explore the relationship between inflation and infant mortality in China.

Recently, research on inflation and population health mainly included two perspectives on food prices and health care prices. On the one hand, numerous studies have shown that inflation increases food prices and poverty in poor areas (17), thereby affecting nutritional access for pregnant women and children and causing a decline in child health. Specifically, Kidane and Andinet (32) found that children under the age of five have about a 5.4 percent lower chance of surviving if they are exposed to a 10% increase in staple food costs while they are still in the womb. Akinlo and Ibrahim. Akinlo and Ibrahim (16) studied the effect of food price increases on under-five and infant mortality in 31 selected sub-Saharan African countries using fixed effects, random effects, differential GMM, and systematic GMM. The results of the study show that food price increases have a significant adverse effect on both infant and under-five mortality in sub-Saharan Africa. Christian (9) finding that in the face of food crises and reduced food availability, rising food prices may have a wide range of consequences for people's nutritional and health status, including enhanced squandering and stunting in children, intrauterine growth restriction, and micronutrient deficiencies like vitamin A, iron, and zinc.

On the other hand, inflation affects the cost of medicine and health care, which in turn has an impact on people's health. Specifically, Cheng (33) argues that the rising cost of health care has become a growing challenge, and this stems from health care inflation. The same view is held by Bayati et al. (34), who argue that the inflation rates lead to reduced access to health care services. Thus, inflation-induced increases in health care prices can indirectly have adverse effects on population health. In addition, Bourne et al. (35) explored the relationship between inflation and mortality and found a small correlation between inflation and mortality. However, the above studies have explored the relationship between inflation and infant mortality using fixed effects, random effects, and other methods. And these traditional methods require all data to be of the same single low-frequency frequency, which can lead to inaccurate estimation results and cannot explore the relationship between the two more deeply. In addition, the above studies mainly focus on extreme poverty in Africa, lacking studies on China, an emerging developing country. Moreover, those researches on inflation mainly focused on food price inflation and health care price inflation, since price inflation affects population health through all aspects of household life, we included the total price index in our study to obtain more comprehensive results.

It is worth noting that higher costs of living and higher food prices do not necessarily increase infant and child mortality. In periods of rising prices, inflation may lead to a decline in real household income. However, as mentioned by Baird et al. (24), it's hard to ascertain how income shocks affect infant mortality. Shocks depress wages, implying a lower opportunity cost of time. Taking children to preventive health care appointments, nursing, making nutritious foods, and gathering safe drinking water are all activities that require a lot of parentals (particularly mothers) attention. Systemic shocks may enhance the prognosis of children by lowering the expense of participating in these activities. Inflation may therefore show a positive correlation to the degree of child health. Indeed, Schultz (36) argues that the value of time is an important determinant of other important events. And Miller et al. (37) point out that when coffee prices are low, parents are more likely to be home rather than working outside the home when their child is sick. Therefore, it is still worth exploring exactly the relationship between inflation and infant mortality.

## Theoretical Analysis and Research Hypothesis

Existing studies have proposed many mechanisms by which inflation can affect infant mortality. The relationship between the two series has been explained in three main ways: income effects, substitution effects, and psychosocial mechanisms. The first perspective focuses on the effect of inflation on infant mortality from the income perspective, which can explain the positive variation of infant mortality with inflation. Specifically, inflation leads to a decline in real household income, which may increase infant mortality. Because families are no longer able to purchase better nutritional or medical care, or even afford the rising prices of medications, resulting in pregnant women not receiving good health care. Moreover, excessive inflation leads to household poverty by reducing their purchasing power, and there is strong evidence that increased child poverty leads to deterioration in child health and increased infant mortality (38, 39). Thus, inflation affects infant mortality through income effects.

The second view focuses on the effect of inflation on infant mortality from the perspective of substitution effects, which could explain that inflation would decrease infant mortality. As proposed by Bhalotra (40), maternal time appears to be an important determinant of the demand for child health inputs. When inflation occurs, the opportunity cost of leisure time decreases as inflation increases. As a result, pregnant women will have more time to spend on health-protective activities and routine medical checkups. More time spent cooking lower-calorie and higher-quality meals at home will result in the intake of more nutrient-rich prepared foods, thereby enhancing infant survival (41). And, when the cost of mothers' time is reduced, mothers will spend more time on their infant's health care, including taking the child for preventive care visits, breastfeeding, cooking healthy foods, and collecting clean water, which may improve the child's prognosis (24). Overall, when such opportunity cost of time is cheap, people, especially pregnant women, will move their time from the labor market to health maintenance activities, at this time, this substitution impact overwhelms the income effect, resulting in a positive net effect on health.

The third perspective explores the impact of psychological factors on infant mortality in the face of inflationary shocks from a psychosocial perspective. Naz et al. (42) suggest that inflation is harming people's psychology to a great extent. It makes people psychologically depressed, creates waves of tension and anxiety, pushes them into turbulent situations. Their inability to buy enough daily goods due to price increases puts them under stress and tension, and if the same process affects pregnant mothers, increased inflation will be associated with increased infant mortality.

Based on the above analysis, we can formulate the following hypotheses:

Hypothesis 1: IMR varies positively with the year-on-year change in CPI, indicating that inflation increases the infant mortality
Hypothesis 2: IMR varies negatively with the year-on-year change in CPI, suggesting that inflation decreases the infant mortality

## Empirical Model

To better investigate the variation of empirical results caused by different sampling frequencies, we introduced the MF-VAR model and LF-VAR model, respectively. The lag length was selected as four to capture the potential seasonality effect as suggested by Motegia and Sadahirob (43), Sun et al. (44), and Liu et al. (2).

All sequences in the LF-VAR model have the same frequency, and when there is inconsistency in sequence frequencies, it is necessary to sum the high-frequency data to the low-frequency data, which may lead to inaccurate estimation results. Ghysels et al. (45) argue that the mixed-frequency Granger causality test can better recover causal patterns in potentially high-frequency processes. Therefore, it is necessary to use the mixed frequency model (MF-VAR) whose expression takes the form of


(1) $$\begin{array}{l} \left[\begin{array}{c} CPI_{1t}\\ CPI_{2t}\\ CPI_{3t}\\ CPI_{4t}\\ IMR_{t}\\ PHE_{t}\\ PCDI_{t} \end{array}\right]=\overset{4}{\underset{k=1}{\sum }}\left[\begin{array}{c} a_{11,k}\;a_{12,k}\;a_{13,k}\;a_{14,k}\;a_{15,k}\;a_{16,k}\;a_{17,k}\\ a_{21,k}\;a_{22,k}\;a_{23,k}\;a_{24,k}\;a_{25,k}\;a_{26,k}\;a_{27,k}\\ a_{31,k}\;a_{32,k}\;a_{33,k}\;a_{34,k}\;a_{35,k}\;a_{36,k}\;a_{37,k}\\ a_{41,k}\;a_{42,k}\;a_{43,k}\;a_{44,k}\;a_{45,k}\;a_{46,k}\;a_{47,k}\\ a_{51,k}\;a_{52,k}\;a_{53,k}\;a_{54,k}\;a_{55,k}\;a_{56,k}\;a_{57,k}\\ a_{61,k}\;a_{62,k}\;a_{63,k}\;a_{64,k}\;a_{65,k}\;a_{66,k}\;a_{67,k}\\ a_{71,k}\;a_{72,k}\;a_{73,k}\;a_{74,k}\;a_{75,k}\;a_{76,k}\;a_{77,k} \end{array}\right]\left[\begin{array}{c} CPI_{1,t-k}\\ CPI_{2,t-k}\\ CPI_{3,t-k}\\ CPI_{4,t-k}\\ IMR_{t-k}\\ PHE_{t-k}\\ PCDI_{t-k} \end{array}\right]+\left[\begin{array}{c} \varepsilon_{1t}\\ \varepsilon_{2t}\\ \varepsilon_{3t}\\ \varepsilon_{4t}\\ \varepsilon_{5t}\\ \varepsilon_{6t}\\ \varepsilon_{7t} \end{array}\right] \end{array}$$

Where *CPI*_*it*_ (*i* = 1, 2, 3, 4) represents the quarterly CPI year-over-year, which is used to indicate the degree of inflation. *IMR*_*t*_ represents the difference in infant mortality rate. Besides, *PHE*_*t*_ and *PCDI*_*t*_ signifies the per capita health care consumption expenditure and the disposable income per capita, respectively. Let *t* ∈ (*i* = 1, 2, 3, 4) denotes each year during the period we study. *a*_*ij,k*_(*i, j* ∈ 1, 2, 3, 4, 5, 6, 7) and *k* ∈ (1, 2, 3, 4) are the elements within the mixed frequency vector autoregression model's parameter matrix. ε_*it*_(*i* = 1, 2, ⋯7) represent error terms.

There are many existing studies on the empirical analysis of MF-VAR (43, 46), and concerning the above studies, to decrease the estimated parameters, we employ demean levels to avoid calculating constants in the parameter matrix. A mixed data sampling regression can reduce the number of coefficients by fitting a function to the parameters of high-frequency variables (43, 47). In Equation (1), we observe that *CPI*_1*t*_, *CPI*_2*t*_, *CPI*_3*t*_, *CPI*_4*t*_ in the form of a vector. Thus, to clarify the link between inflation and infant mortality rate more clearly, Equation (1) can be rewritten as:


(2) $$IMR_{t}=\sum_{k=1}^{4}[\sum_{j=1}^{4}a_{5j,k}CPI_{j,t-k}+a_{55,k}IMR_{t-k}+a_{56,k}PHE_{t-k}+a_{57,k}PCDI_{t-k}]+\varepsilon_{5t}$$

Different values *a*_5*j,k*_ can be taken under *j* = 1, 2, 3, 4. And it's important to note that the year-over-year CPI growth rate in each quarter (*CPI*_1*t*_, *CPI*_2*t*_, *CPI*_3*t*_, *CPI*_4*t*_) has a different effect on the infant mortality rate, that is, quarterly inflation has a heterogeneous effect on infant mortality.

We introduced the LF-VAR model to establish a comparison between the VAR model with mixed frequency data and the classic VAR model with single frequency data, and the LF-VAR expressed as follows:


(3) $$\begin{array}{l} \left[\begin{array}{c} CPI_{t}\\ IMR_{t}\\ PHE_{t}\\ PCDI_{t} \end{array}\right]=\overset{4}{\underset{k=1}{\sum }}\left[\begin{array}{c} a_{11,k}\;a_{12,k}\;a_{13,k}\;a_{14,k}\\ a_{21,k}\;a_{22,k}\;a_{23,k}\;a_{24,k}\\ a_{31,k}\;a_{32,k}\;a_{33,k}\;a_{34,k}\\ a_{41,k}\;a_{42,k}\;a_{43,k}\;a_{44,k} \end{array}\right]\left[\begin{array}{c} CPI_{t-k}\\ IMR_{t-k}\\ PHE_{t-k}\\ PCDI_{t-k} \end{array}\right]+\left[\begin{array}{c} \varepsilon_{1t}\\ \varepsilon_{2t}\\ \varepsilon_{3t}\\ \varepsilon_{4t} \end{array}\right] \end{array}$$

Where *CPI*_*t*_ refers to the annual CPI year-over-year growth rate, which is the average of the four quarterly CPI year-over-year growth rates, and is used to measure the annual level of inflation. The remaining elements are defined in the same way as the elements in Equation (1). Similarly, as in Equation (2), the relationship between inflation and infant mortality can be written in the following form:


(4) $$IMR_{t}=\sum_{k=1}^{4}(a_{21,k}CPI_{t-k}+a_{22,k}IMR_{t-k}+a_{23,k}PHE_{t-k}+a_{24,k}PCDI_{t-k})+\varepsilon_{2t}$$

Equation (4) implies that the year-on-year CPI growth rates in the four quarters (*CPI*_1*t*_, *CPI*_2*t*_, *CPI*_3*t*_, *CPI*_4*t*_) have a homogeneous effect on infant mortality. And The LF-VAR model excludes seasonal effects. Then let *CPI*_*t*_ = (*CPI*_1*t*_ + *CPI*_2*t*_ + *CPI*_3*t*_ + *CPI*_4*t*_)/4, in which *CPI*_*it*_ denotes the year-over-year CPI at the *ith* quarter of year t, where *i* = 1, 2, 3, 4. Equation (4) could be further extended to Equation (5)


(5) $$IMR_{t}=\sum_{k=1}^{4}[a_{21,k}(\frac{1}{4}\sum_{i=1}^{4}CPI_{i,t-k})+a_{22,k}IMR_{t-k}+a_{23,k}PHE_{t-k}+a_{24,k}PCDI_{t-k}]+\varepsilon_{2t}$$

In General, we aggregate time series of different frequencies to a common lowest frequency by time summation to test for causality between the series. However, it has been shown that the way of turning high-frequency variables into low-frequency variables by time summation is too coarse and can lead to inaccurate statistical results (48). Such problems can be well-avoided by the MF-VAR model, which does not involve any filtering procedure and can retrieve causal patterns in potentially high-frequency processes more effectively. In addition, for local alternatives, the mixed-frequency causality test has stronger asymptotic power and can capture the heterogeneous effects of high-frequency series on low-frequency variables (49). As a result, we choose the MF-VAR model to capture the effect of quarterly CPI year-over-year growth on infant mortality.

In the following content, each model is subjected to impulse response analysis and forecast error variance decomposition in this research. We follow Liu et al. (2), and according to the economic principles of Income effect and substitution effect, we set the Cholesky order for the LF-VAR and MF-VAR models, which are *CPI*_*t*_ → *IMR*_*t*_ → *PHE*_*t*_ → *PCDI*_*t*_ and *CPI*_1*t*_ → *CPI*_2*t*_ → *CPI*_3*t*_ → *CPI*_4*t*_ → *PHE*_*t*_ → *PCDI*_*t*_ respectively.

## Data and Variables

The primary goal of this research is to look into the relationship between inflation and infant mortality. To eliminate seasonal factors, we retrieved the year-on-year consumer price index (CPI) from the first quarter of 1999 to the fourth quarter of 2019 in China to measure inflation. Also, we select the mortality rate per 1,000 newborns in China from 1999 to 2019 as the infant mortality rate which is annual data. All data above are obtained from the Wind database. We choose these study periods because they are the longest series of data available for all the variables we are analyzing.

**Figure 1:**
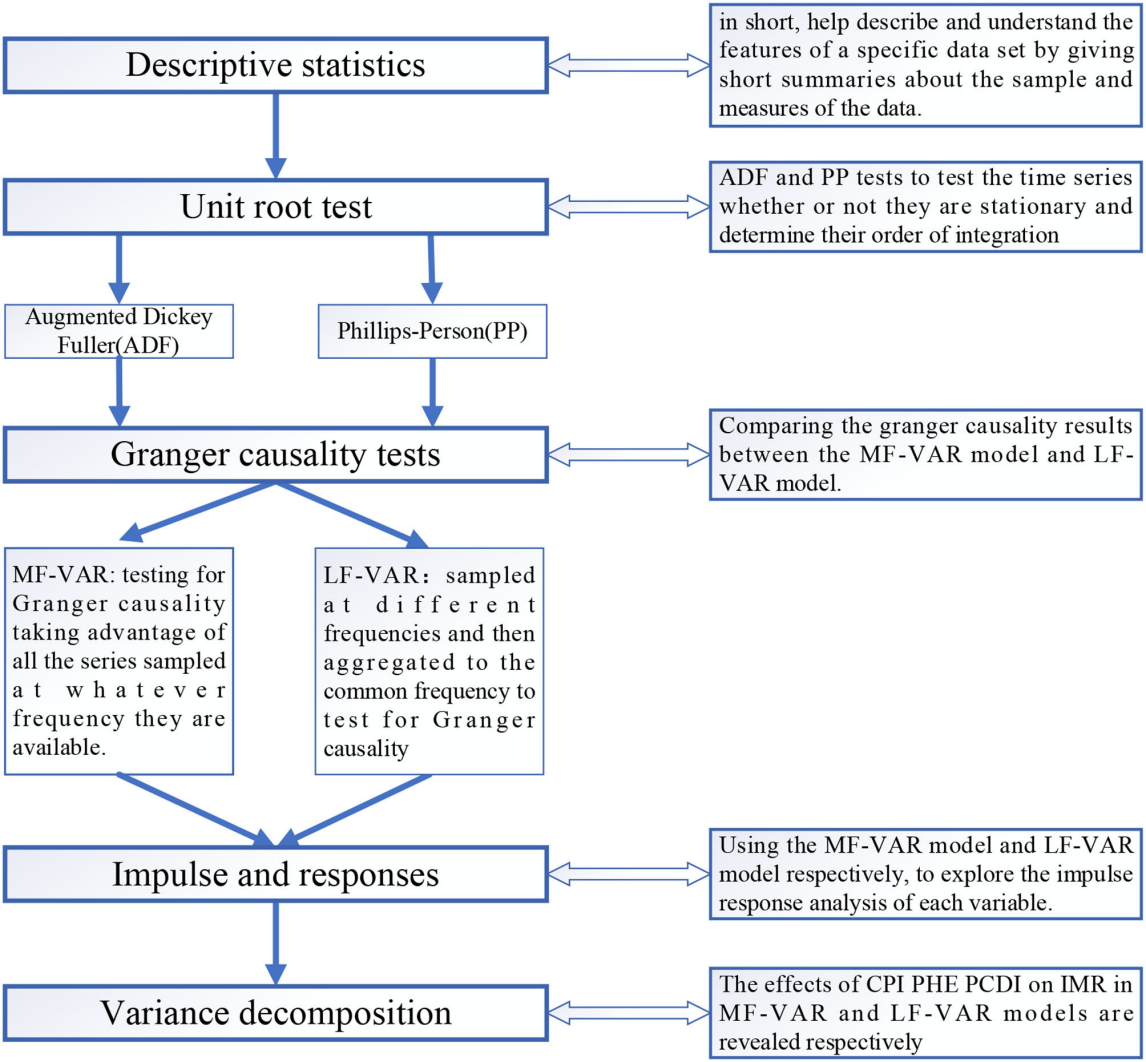
Flowchart of the data analysis.

There is considerable research suggesting that there is a negative relationship between health expenditure and infant mortality rates (50) and that the suppressive effect of per capita health consumption expenditure on infant mortality rates is more pronounced at high development stages. So, we included per capita health consumption (measured as the annual growth rate of per capita health consumption expenditure.) as a control variable to prevent possible confounding effects, which might create a spurious correlation between inflation and infant mortality rates. Previous studies on infant mortality have found a causal relationship between income and infant mortality (51), so the second control variable incorporated in the model is the per capita disposable income, which reflects the standard of living of its citizens. More specifically, Nwude et al. (52) indicated that in underdeveloped countries, income per capita improves health outcomes dramatically. Zilidis et al. (53) argue that long-term income reductions harm infant mortality. Followed by Liu et al. (2) PHE and PCDI are measured by annual growth rate per capita, that is, they are computed as the difference between the logarithm of the variable at the t-th year and that from the previous year. Data above all were all range from 1999 to 2019 and are obtained from the Wind database. And we provide a flowchart of the data analysis to better show the research process, which shows in Figure 1.

## Results

### Descriptive Statistics

Table 1 presents the summary statistics of the quarterly year-over-year values of consumer price index (CPI), infant mortality rate (IMR), the growth rate of per capita health expenditure (PHE), and growth rates of national per capita income (PCDI) ranging from 1999 to 2019. From Table 1, we can see that the mean and standard deviation of the CPI for the first and second quarters are not significantly different, and much higher than that of the third and fourth quarters. From the perspective of skewness, the CPI in the third and fourth quarters and the PHE are negatively skewed. Which suggests that these variables are left-skewed. All other series are positively skewed, that is right-skewed. Except for variables of IMR, and PCDI, all series have kurtosis values greater than three, implying that the distribution is leptokurtic. Besides, the CPI series in the second quarter and the PCDI are normally distributed, while the rest of the variables are non-normally distributed at the 1% significance level. However, Ghysels et al. (45) say that the MF-VAR model does not require the normality assumption. To ensure the stationary of the series for better use in the MF-VAR and LF-VAR models, we take the ADF with PP method to test the stationary of the series, and the results are shown in Table 2. It can be seen that all the series reject the original hypothesis and prove that the series is the station, except for the infant mortality, and per capita income. So, we performed the first-order difference of those variables. Then the above variables can be used in the LF-VAR and MF-VAR models.

**Table 1:** Descriptive statistics.

	**CPI1**	**CPI2**	**CPI3**	**CPI4**	**CPIA**	**IMR**	**PHE**	**PCDI**
Mean	1.938	1.995	0.162	0.320	1.103	16.548	0.104	0.107
Median	2.134	1.833	0.178	0.267	1.130	14.700	0.121	0.100
Maximum	8.033	7.767	0.689	0.978	4.370	31.900	0.202	0.172
Minimum	−1.433	−2.167	−1.167	−0.833	−1.400	6.800	−0.346	0.066
Std. Dev.	2.070	2.279	0.345	0.394	1.167	7.933	0.108	0.028
Skewness	1.053	0.477	−2.612	−0.699	0.637	0.539	−3.616	0.610
Kurtosis	4.923	3.714	11.925	4.865	4.885	2.036	15.811	1.422
Jarque-Bera	7.118	1.241	93.575	4.754	4.527	1.832	189.372	0.491
Probability	0.029	0.538	0.000	0.093	0.104	0.400	0.000	0.722

**Table 2:** Unit root test.

	**ADF unit root tests**			**PP unit root tests**		
	**Intercept**	**Intercept and trend**	**None**	**Intercept**	**Intercept and trend**	**None**
CPI1	−4.099^***^	−3.555^**^	−0.526	−4.100^***^	−4.064^**^	−2.226^**^
CPI2	−3.841^***^	−3.783^**^	−0.505	−4.651^***^	−4.558^***^	−2.404^**^
CPI3	−8.864^***^	−8.740^**^	−4.675^**^	−8.402^***^	−8.740^***^	−4.390^***^
CPI4	−6.603^***^	−7.447^***^	−3.114^***^	−36.048^***^	−7.447^***^	−3.234^***^
CPIA	−4.543^***^	−4.388^**^	−0.467	−4.577^***^	−4.393^**^	−2.188^**^
IMR	−13.504^***^	−0.460	−35.351^***^	−10.258^***^	−0.460	−27.037^***^
PHE	−3.723^**^	−3.709^*^	−6.139^***^	−4.575^***^	−4.877^***^	−2.756^***^
PCDI	−2.085	−1.773	−0.319	−2.137	−2.058	−0.133
DIMR	−4.661^***^	−4.601^***^	−1.484^***^	−4.731^***^	−4.590^***^	−3.845^***^
DPCDI	−5.112^***^	−6.088^***^	−5.285^***^	−4.690^***^	−6.401^***^	−4.873^***^

*** *Represents 1% significance level*.

** *Represents 5% significance level*.

* *Represents 10% significance level*.

### Granger Causality Tests

We present the test results of Granger causality under the MF-VAR model and LF-VAR model, respectively. As shown in Table 3, we find a single granger causal relationship between inflation and infant mortality in China from 1999 to 2019, and we reject the null hypothesis that there is a non-causal relationship from inflation to infant mortality (*CPI* ≠> Δ*IMR*) at the 5% significance level, that is inflation affects infant mortality. The causal relationship between inflation and infant mortality is consistent with the relationship based on food prices and infant mortality studied by Kidane and Andinet (32), in which higher food prices reduce the survival of Children under five. In addition, we similarly reject the original hypothesis of a non-causal relationship between inflation and per capital expenditure (*CPI* ≠> *PHE*) between 1999 and 2019, as well as the non-causal relationship between inflation and the per capita disposable income (*CPI* ≠> Δ*PCDI*). Moreover, the LF-VAR model does not find a causal relationship between the variables, which may reflect the strength of the MF-VAR model. Compared with the LF-VAR model, the VAR model has higher explanatory power and better reveals causal relationships in the generation of potentially high-frequency data (45).

**Table 3:** Granger causality test.

**MF-VAR**		**LF-VAR**	
**Null hypothesis**	***p*-value**	**Null hypothesis**	***p*-value**
Δ*IMR* ≠> *CPI*	0.5175	Δ*IMR* ≠> *CPI*	0.442
*PHE* ≠> *CPI*	0.075^**^	*PHE* ≠> *CPI*	06815
Δ*PCDI* ≠> *CPI*	0.646	Δ*PCDI* ≠> *CPI*	0.5275
*CPI* ≠> Δ*IMR*	0.038^**^	*CPI* ≠> Δ*IMR*	0.1375
*PHE* ≠> Δ*IMR*	0.303	*PHE* ≠> Δ*IMR*	0.374
Δ*PCDI* ≠> Δ*IMR*	0.439	Δ*PCDI* ≠> Δ*IMR*	0.4585
*CPI* ≠> *PHE*	0.033^**^	*CPI* ≠> *PHE*	0.238
Δ*IMR* ≠> *PHE*	0.8035	Δ*IMR* ≠> *PHE*	0.611
Δ*PCDI* ≠> *PHE*	0.8375	Δ*PCDI* ≠> *PHE*	0.8355
*CPI* ≠> Δ*PCDI*	0.0145^**^	*CPI* ≠> Δ*PCDI*	0.2405
Δ*IMR* ≠> Δ*PCDI*	0.724	Δ*IMR* ≠> Δ*PCDI*	0.41
*PHE* ≠> Δ*PCDI*	0.941	*PHE* ≠> Δ*PCDI*	0.4215

** *Represents 5% significance level*.

### Impulse and Variance Decomposition

**Figure 2:**
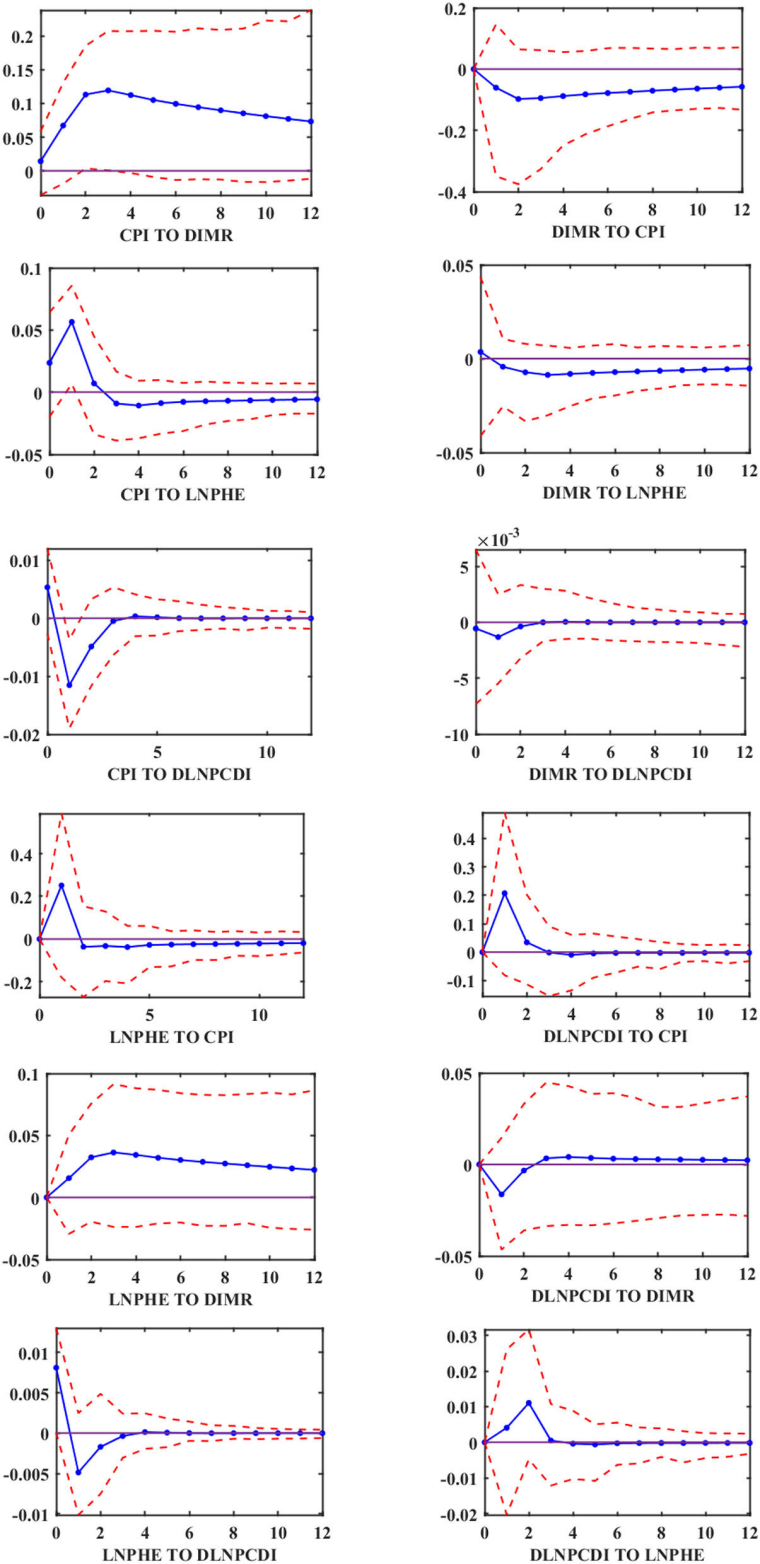
Impulse response functions of LF-VAR.

We find a single directional Granger causal effect from inflation to infant mortality in China throughout 1999–2019. To explore the relationship between them in more detail, we use the MF-VAR model and LF-VAR model, respectively, to explore the impulse response analysis of each variable. The empirical results of the LF-VAR model are analyzed in the first stage. The impulse response functions at 95% confidence intervals are reported in Figure 2. It can be noted that the response of infant mortality to positive shock from inflation (denoted as “CPI TO DIMR”) is significantly positive during a 12-year horizon, which suggests that infant mortality rises in response to inflation shock. We can also see that the per capita health care expenditure under the shock of inflation is significantly positive until the second-year horizon and becomes negative thereafter. The disposable income per capita is significantly negative in response to the shock of inflation, and there is no significant impact from inflation on the disposable income per capita after the fourth-year horizon.

**Figure 3:**
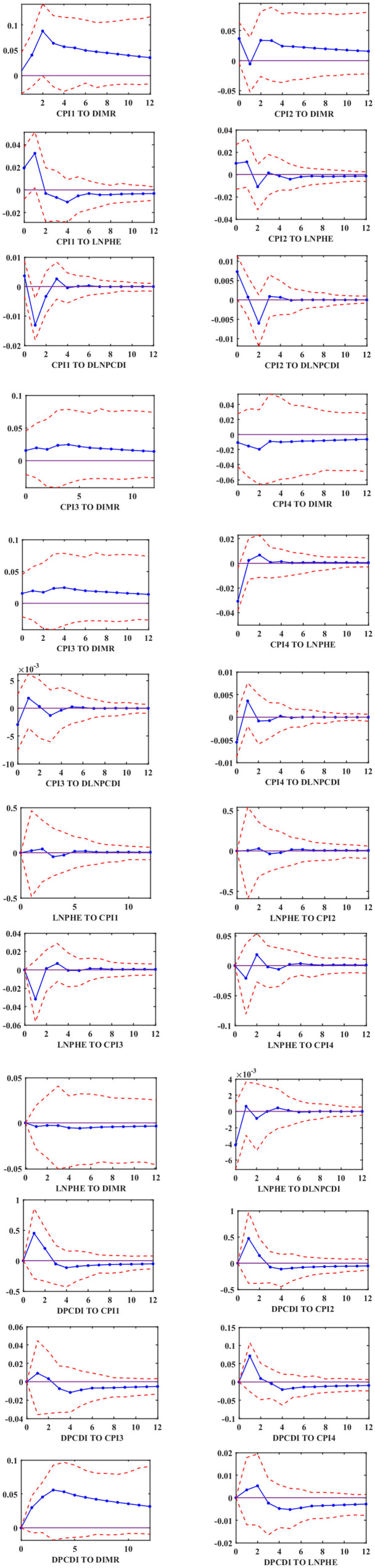
Impulse response functions based on MF-VAR.

In the second stage, the empirical results of the MF-VAR model are examined. The impulse response functions at 95% confidence intervals are reported in Figure 3. we can see that the response of infant mortality to inflation shocks (“CPI TO DIMR”) in the first, second, and third quarters is significantly positive in any horizon, while the effect of inflation on infant mortality in the fourth quarter is significantly negative in the horizon 0–12. It demonstrates that the MF-VAR model has greater explanatory ability than other traditional VAR models and explains why the relationship between inflation and infant mortality is variant. As for the relationship between inflation and the growth rate of per capita health care expenditure, we find that the response of the per capita health care expenditure to inflation shocks in the first and second quarters is significantly positive in the first two periods and starts to be significantly negative after the second period. While the situation is roughly opposite in the third and fourth quarter. Besides, the disposable income per capita in the face of inflationary shocks in the first and second quarters is first significantly negative, then significantly positive, and ceases to be significant after two horizons. In the third and fourth quarters, the disposable income per capita turns from negative to positive in the face of inflationary shocks and gradually becomes insignificant after two periods.

Tables 4, 5 show the forecast error variance decomposition for the LF-VAR and MFVAR models, respectively. We perform variance decomposition for each variable in the short-run (h = 1), medium-run (h = 4), long-run (h = 8), and very long-run (h = 12), respectively. As shown in Table 4, in the LF-VAR model, the proportions of forecast error variance of Infant mortality (IMR) attributed to inflation (CPI) in the LF-VAR is 8.5, 31.7, 32.4, and 32.8 when h = 1, h = 4, h = 8, and 12, respectively. While in the MF-VAR model, the proportions of forecast error variance of Infant mortality (IMR) attributed to total CPI from each quarterly CPI (∑*CPI*_*i*_) is 19.7 45.3, 44.9, and 44.6 in the corresponding horizons, respectively. We can see that the contribution degree of forecast error variance of inflation on infant mortality in the MF-VAR model is 2.32, 1.43, 1.39, and 1.36 times higher than that in the LF-VAR model, when h = 1, h = 4, h = 8, and 12, respectively. Similarly, the proportion of prediction error variance attributable to inflation (CPI) for the growth rate of per capita health care expenditure (PHE) is 2.0, 15.9, 16.0, and 16.1 in the LF-VAR model in different periods (short, medium, long, and very long term), respectively. While, in the MF-VAR model, the data is 23.3, 42.9, 43.1, and 43.1 in the corresponding horizons, respectively. the contribution is 11.65, 2.70, 2.69, and 2.68 times higher than that of the LF-VAR model in the corresponding periods. As for the per capita disposable income, In the LF-VAR model, the forecast error variance attributed to CPI is 5.0, 23.6, 23.9, 24.0 with the period continues to increase, while in the MF-VAR model, the values are 3.66, 2.42, 2.42, 2.41 times higher in the corresponding horizons, respectively.

**Table 4:** Forecast error variance decomposition of LF-VAR.

	**CPI**	**DIMR**	**PHE**	**PCDI**
**Decomposition of CPI**				
h = 1	84.2	7.9	2.2	5.7
h = 4	70.2	6.8	5.7	17.3
h = 8	69.7	7.3	5.8	17.2
h = 12	69.3	7.8	5.9	17.0
**Decomposition of DIMR**				
h = 1	8.5	89.7	1.5	0.3
h = 4	31.7	55.7	8.1	4.5
h = 8	32.4	54.4	8.3	4.8
h = 12	32.8	53.9	8.5	4.9
**Decomposition of PHE**				
h = 1	2.0	1.3	76.2	20.5
h = 4	15.9	2.2	62.3	19.6
h = 8	16.0	2.3	62.1	19.6
h = 12	16.1	2.4	62.0	19.6
**Decomposition of PCDI**				
h = 1	5.0	0.3	20.1	74.6
h = 4	23.6	3.4	20.2	52.7
h = 8	23.9	4.1	20.0	52.0
h = 12	24.0	4.7	19.9	51.4

**Table 5:** Forecast error variance decomposition of MF-VAR.

	**CPI1**	**CPI2**	**CPI3**	**CPI4**	**SUM (*CPI*_*i*_)**	**DIMR**	**PHE**	**PCDI**
**Decomposition of CPI1**								
h = 1	40.7	37.8	10.0	7.2	95.7	4.0	0.0	0.3
h = 4	34.4	32.3	10.9	6.1	83.7	4.3	0.2	11.8
h = 8	34.3	32.3	10.8	6.1	83.5	4.5	0.2	11.8
h = 12	34.2	32.3	10.8	6.1	83.4	4.6	0.2	11.9
**Decomposition of CPI2**								
h = 1	37.2	40.0	5.2	8.4	90.8	7.2	0.1	1.9
h = 4	34.1	35.5	7.4	7.3	84.3	6.2	0.6	8.9
h = 8	34.0	35.4	7.4	7.3	84.1	6.3	0.6	9.1
h = 12	34.0	35.4	7.4	7.3	84.1	6.3	0.6	9.1
**Decomposition of CPI3**								
h = 1	14.3	7.6	58.1	6.7	86.7	0.8	4.7	14.3
h = 4	16.9	10.7	48.4	6.3	82.3	1.6	7.3	8.9
h = 8	17.1	11.0	47.8	6.2	82.1	1.8	7.2	8.9
h = 12	17.1	11.0	47.5	6.2	81.8	2.1	7.2	9.0
**Decomposition of CPI4**								
h = 1	9.7	11.6	6.4	54.9	82.6	0.1	14.6	2.8
h = 4	11.6	13.6	7.1	43.3	75.6	4.2	12.3	7.9
h = 8	11.9	13.8	7.3	41.2	74.2	5.4	11.7	8.7
h = 12	12.0	13.9	7.3	40.0	73.2	6.5	11.4	8.9
**Decomposition of IMR**								
h = 1	6.6	12.1	0.9	0.1	19.7	67.1	9.5	3.7
h = 4	17.4	20.5	5.9	1.5	45.3	41.1	2.8	10.9
h = 8	16.9	20.0	6.4	1.6	44.9	41.1	1.9	12.2
h = 12	16.7	19.9	6.4	1.6	44.6	41.0	1.7	12.7
**Decomposition of PHE**								
h = 1	0.0	0.1	5.4	17.8	23.3	9.4	66.6	0.7
h = 4	6.2	10.2	9.5	17.0	42.9	13.9	40.1	3.0
h = 8	6.3	10.3	9.6	16.9	43.1	13.9	40.0	3.0
h = 12	6.3	10.3	9.6	16.9	43.1	13.9	40.0	3.0
**Decomposition of PCDI**								
h = 1	0.5	3.6	10.3	3.9	18.3	4.3	0.8	76.7
h = 4	22.8	22.5	8.9	2.8	57.0	5.8	1.2	35.8
h = 8	23.1	22.6	9.4	2.8	57.9	6.0	1.3	34.8
h = 12	23.1	22.7	9.3	2.8	57.9	6.2	1.3	34.7

According to the previous research we have done, we can find that, on the one hand, the forecast error variance decomposition of infant mortality confirms the large explanatory power of inflation, indicating that inflation affects the level of health of the population, and combined with Figure 2 we can know that infant mortality increases under the impact of inflation. On the other hand, we find a positive causal relationship between inflation and infant mortality in the MF-VAR model, while causality relationships between them are not identified in the LF-VAR model. Moreover, In the MF-VAR model, the forecast error variance decomposition of infant mortality attributed to inflation is higher than the value calculated in the LF-VAR model. This is because the LF-VAR model has comparatively low interdependence among the variables, but the MF-VAR model has relatively high interdependence among variables, and it has more explanatory power in investigating the relationship between inflation and infant mortality than the LF-VAR model.

## Discussion

We analyzed the relationship between inflation and infant mortality using MF-VAR and LF-VAR models, from the mixed-frequency causal effects tests, impulse response analysis, and forecast error variance decomposition performed yielded three important conclusions. Firstly, the relationship between inflation and infant mortality is variant. Figure 3 reveals the positive and negative relationship between inflation and infant mortality, respectively. With a positive correlation between CPI and infant mortality in each of the first three quarters, namely, inflation leads to an increase in infant mortality, and a negative correlation in the fourth quarter, which means that inflation leads to a decrease in infant mortality. Previous studies on the effect of inflation on infant mortality have found positive (9, 17, 31) or negative (21, 34) correlations between inflation and infant mortality, and varied across time. In contrast, by introducing high-frequency data into time series analysis utilizing MF-VAR models, we can effectively reconcile these previously contradictory results.

Secondly, our results provide a good test of our hypothesis 1, that inflation increases infant mortality. This is because inflation leads to an increase in the cost of goods or services and a decrease in real wages, which triggers a decline in household living standards (54), The rising cost of living affects infant mortality through all aspects of life, including food prices and health care prices and so on. Specifically, inflation invisibly raises food prices, causing households to shift spending from more expensive sources of protein, such as meat, fish, and eggs, to relatively inexpensive grains. This leads to vitamin deficiencies and even malnutrition among pregnant and lactating women, increasing the risk of infant mortality (16). Moreover, maternal malnutrition caused by rising food prices may also lead to poor birth outcomes, such as fetal growth restriction and preterm delivery, which can also trigger an increase in infant mortality (9, 17, 54, 55). On the other hand, inflation leads to an increase in health care costs, thus making high health care costs unaffordable for some families or reducing health care expenditures, making families less healthy, which may affect infant mortality. Therefore, we can conclude that the rise in inflation leads to an increase in infant mortality.

At the same time, the causality results lead to the confirmation of the income effect and the substitution effect. As shown in Table 3, there is a causal relationship between inflation and infant mortality, that is, inflation reduces the quality of life of families by increasing their cost of living, which in turn leads to higher infant mortality. However, inflation does not increase infant mortality in all periods, as shown in Figure 3, where there is a negative correlation between inflation and infant mortality (CPI4 TO DIMR) in the fourth quarter. Inflation acts on infant mortality through income effects and substitution effects. Inflation not only increases the substitution effect of maternal health maintenance activities by reducing the opportunity cost of self-health protection activities relative to productive activities, but also reduces the income effect by increasing household cost, which means that inflation invariably increases expenditures on daily necessities such as food and medical care, and also reduces the ability to maintain health, invariably reducing pregnant women's and infants' access to health care and other goods and services. In previous studies, many studies suggest that recessionary inflation leads to a substitution effect that is superior to the income effect (27, 37, 56), thus contributing to the reduction in infant mortality. However, when we consider the effect of the aggregate effect of inflation (the inflation rate in the four quarters as a whole) on infant mortality. The significant negative relationship between the two in the fourth quarter is offset by a positive effect in the first three quarters. These findings suggest that the income effect outweighs the substitution effect in the relationship between inflation and infant mortality. Inflation-induced reductions in relative income have a more severe impact on infant mortality, the result that also supports Hypothesis 1: inflation increases infant mortality.

Finally, the causal relationship between inflation and infant mortality can be obtained by taking different frequency time series into account through the MF-VAR model, while the causal relationship cannot be observed by aggregating multiple frequency time series into the lowest frequency data through the LF-VAR model, indicating that the MF-VAR model has better explanatory power than the LF-VAR model. Moreover, from the results of the prediction error variance decomposition in Tables 4, 5, we can also see that the contribution of inflation to the prediction error variance of infant mortality in the MF-VAR model is 46.6 at h=12 instead of 32.8 in the LF-VAR model, indicating that the contribution of inflation to infant mortality is much higher than that measured by the traditional model. The contribution degree of forecast error variance of inflation on infant mortality in the MF-VAR model is 2.32, 1.43, 1.39, and 1.36 times higher than that in the LF-VAR model, in different horizons (h = 1, h = 4, h = 8, and h = 12, respectively). This also explains the weak effect of inflation on infant mortality that has been previously reported (9). And it can be found that the model including high-frequency data provides a more thorough picture of the relationship between both inflation and infant mortality compared to previous studies.

## Conclusion

Infant mortality is a truly critical indicator of social and economic development in all countries, and reducing neonatal mortality is an important goal of the Sustainable Development Goals (SDGs). Many factors affect infant mortality, and in this paper, we focus on the causality between inflation among the macroeconomic indicators and infant mortality in China. This is because, inflation is considered one of the issues of concern in the current situation, and excessive price increases may bring tragedy to the general public. And with the global inflation severe caused by the COVID-19, it is extremely important to explore the relationship between inflation and infant mortality. Therefore, this paper investigates the relationship between inflation and infant mortality in China using the MF-VAR model that does not require any filtering procedure. The heterogeneous effect of quarterly inflation on infant mortality is explored using quarterly CPI growth rates year-on-year as well as annual infant mortality rates.

We find that a causal relationship between inflation and infant mortality can be detected using the MF-VAR model, indicating that more accurate and comprehensive results can be obtained using mixed-frequency data. In addition, in the quarterly data, inflation increases infant mortality in the first three quarters and inflation decreases IMR in the fourth quarter, but overall, the income effect outweighs the substitution effect, leading to an increase in infant mortality due to total inflation. Moreover, compared with the LF-VAR model, the contribution of CPI to IMR is greater in the forecast error variance decomposition of the MF-VAR model, and the quarterly CPI in the mixed frequency model has stronger explanatory power for the annual IMR, indicating that the aggregation of quarterly CPI year-over-year growth rates to annual values in the LF-VAR model underestimates the effect of inflation on IMR.

Understanding the connection between inflation and infant mortality has crucial implications for China and other developing countries. the findings highlight that inflation leads to higher costs of living for households and thus has a profound impact on infant mortality. Since infant survival in China depends to some extent on changes in the relative prices of everyday goods, this finding can help in the development of targeted policies, which implies that the inquiry into how to reduce infant mortality requires that attention must be focused on inflation as a macroeconomic variable. Specifically, during periods of high price increases for goods, the government is supposed to develop targeted programs that focus on stabilizing the prices of food and drug and medical health care. And while stabilizing prices, provide relevant nutritional support programs for pregnant women, breastfeeding mothers, and infants. In addition, the government could increase public spending on health care in times of inflation, thereby reducing the pressure on households to spend on health care.

## Data Availability Statement

The original contributions presented in the study are included in the article/supplementary material, further inquiries can be directed to the corresponding author/s.

## Author Contributions

X-yL: conceptualization, methodology, software, data curation, and writing—original draft preparation. WJ: writing-reviewing and editing. All authors contributed to the article and approved the submitted version.

## Funding

This research was supported by the National Social Science Foundation of China (No. 20BJL020).

## Conflict of Interest

The authors declare that the research was conducted in the absence of any commercial or financial relationships that could be construed as a potential conflict of interest.

## Publisher's Note

All claims expressed in this article are solely those of the authors and do not necessarily represent those of their affiliated organizations, or those of the publisher, the editors and the reviewers. Any product that may be evaluated in this article, or claim that may be made by its manufacturer, is not guaranteed or endorsed by the publisher.
